# MicroRNA-26a Inhibits Angiogenesis by Down-Regulating VEGFA through the PIK3C2α/Akt/HIF-1α Pathway in Hepatocellular Carcinoma

**DOI:** 10.1371/journal.pone.0077957

**Published:** 2013-10-23

**Authors:** Zong-Tao Chai, Jian Kong, Xiao-Dong Zhu, Yuan-Yuan Zhang, Lu Lu, Jia-Min Zhou, Long-Rong Wang, Ke-Zhi Zhang, Qiang-Bo Zhang, Jian-Yang Ao, Miao Wang, Wei-Zhong Wu, Lu Wang, Zhao-You Tang, Hui-Chuan Sun

**Affiliations:** 1 Liver Cancer Institute, Zhongshan Hospital, Fudan University; Key Laboratory of Carcinogenesis and Cancer Invasion (Fudan University), Ministry of Education, Shanghai, P.R. China; 2 Department of Hepatobiliary Surgery, Capital Medical University, Beijing Chaoyang Hospital, Beijing, P.R. China; Institut für Pathologie, Greifswald, Germany, Germany

## Abstract

**Background & Aims:**

microRNAs (miRNAs) have been reported to regulate angiogenesis by down-regulating the expression of pro-angiogenic or anti-angiogenic factors. The aims of this study were to investigate whether miR-26a inhibited angiogenesis by down-regulating vascular endothelial growth factor A (VEGFA) and its clinical relevance in hepatocellular carcinoma (HCC).

**Methods:**

The expression of miR-26a was modified in HepG2 and HCCLM3 cell lines respectively, and a panel of angiogenic factors was measured by real-time PCR in the cells. A luciferase reporter assay was used to validate the target gene of miR-26a. Specific inhibitors of signal transduction pathway and siRNA approaches were used to explore the regulatory mechanism of miR-26a. Migration and tube forming assays were conducted to show the changes of angiogenesis induced by miR-26a and its target genes. Finally animal studies were used to further validate those findings.

**Results:**

Ectopic expression of miR-26a exhibited decreased levels of VEGFA in HepG2 cells. Migration and tube forming of human umbilical vein endothelial cells (HUVECs) were decreased in the conditioned medium from ectopic expression of miR-26a in HepG2 cells compared to control HepG2 cells. The pro-angiogenic effects of the conditioned medium of HepG2 cells on HUVECs were specifically decreased by LY294002, YC-1, and bevacizumab. Integrated analysis disclosed PIK3C2α as a downstream target gene of miR-26a. Ectopic expression of miR-26a suppressed ectopic and orthotopic tumor growth and vascularity in nude mice. The results in HCCLM3 were consistent with those in HepG2. miR-26a expression was inversely correlated with VEGFA expression in HCC patients.

**Conclusions:**

miR-26a modulated angiogenesis of HCC through the PIK3C2α/Akt/HIF-1α/VEGFA pathway. The expression of VEGFA was inversely correlated with miR-26a expression in HCC tumors.

## Introduction

Hepatocellular carcinoma (HCC) is the sixth most common cancer and third-leading cause of cancer-related death worldwide [1]. HCC is characterized by hyper-vascularity, which suggests a crucial role for angiogenesis in tumor development. Hepatic resection and transplantation have been considered as the main curative therapy, but tumor recurrence rate prevents long-term survival [2]. Sorafenib, an inhibitor of multiple kinases including Raf-1 and vascular endothelial growth factor (VEGF) receptor, can be used to inhibit angiogenesis in HCC and has shown benefits in patients with advanced HCC; however, the efficacy is modest [3,4]. Therefore, more effective therapy remains to be developed.

microRNAs (miRNAs) are a class of 22-nucleotide noncoding RNAs that are evolutionarily conserved and function as negative regulators of gene expression. miRNAs down-regulate gene expression by either inducing degradation of target mRNAs or affecting their translation by binding to the 3′ untranslated regions (UTRs) of mRNAs. miRNAs are involved in many biological events, including cell growth, differentiation, apoptosis, fat metabolism, and viral infection [5]. They also play an important role in many types of human cancers [6], and aberrant expression of specific miRNA is directly implicated in tumorigenesis including growth, apoptosis, metastasis, and especially angiogenesis [7–10]. miRNAs have been reported to regulate angiogenesis by down-regulating the expression of pro-angiogenic or anti-angiogenic factors [11–14]. VEGFA is the most potent pro-angiogenesis factor, acting directly on endothelial cells to induce endothelial cell proliferation, migration, survival, and finally angiogenesis, which facilitates tumor growth. VEGFA expression can be mediated by the PI3K/Akt/HIF-1α signaling pathway. miR-15a, miR-16, and miR-503 were reported to inhibit tumor angiogenesis by targeting VEGFA [15],[16]. PIK3C2α, which belongs to class II PI3Ks and can recruit Akt by phosphorylating PIP_2_, has an essential role in angiogenesis. Yoshioka et al. found knockdown of PIK3C2α inhibited VEGFA-induced capillary-like tube formation and transwell migration toward VEGFA in human umbilical vein endothelial cells (HUVECs) [17]. Biswas et al. also found that PIK3C2α was essential for S1P_1_-induced S1P_1_ internalization, endosomal Rac1 activation, and cell migration in endothelial cells [18]. But whether the PIK3C2α/Akt/VEGFA signaling pathway is regulated by miRNAs is still not clear.

Deregulation of miR-26a may differ according to the type of cancer, and miR-26a may play a dual role in tumorigenicity, functioning either as a tumor suppressor or promoter. Studies have found miR-26a is down-regulated in various tumor types such as breast cancer, oral squamous cell carcinoma, and anaplastic carcinomas [19–22], but up-regulated in glioma and cholangiocarcinoma [23–25]. Our previous study found that HCC tumors had a reduced level of miR-26a compared with paired noncancerous liver tissues, and patients with low miR-26a expression had shorter overall survival [26]. In addition, another study in our institute found that patients with hepatitis B virus–related HCC had a lower level of miR-26a in blood compared with patients with chronic hepatitis B [27]. Systemic administration of miR-26a in an HCC mouse model inhibited cancer cell proliferation and provided dramatic protection from disease progression without toxicity [28–30]. All the data suggested miR-26a could present an anti-tumor effect in HCC. Potential mechanisms of miR-26a in HCC could include induction of cell-cycle arrest by direct targeting cyclin D2 and E2 [28] or through the IL-6-stat3 signaling pathway [31]. However, no studies have been done to address the role of miRNA-26a in HCC angiogenesis. 

In the present study, we investigated whether miR-26a expression inhibits angiogenesis in HCC and explored its underlying mechanisms. 

## Materials and Methods

### Cell Lines

The human HCC cell lines HepG2 (Shanghai Institute of Cell Biology, Shanghai, China) and HCCLM3 [32] (Liver Cancer Institute, Fudan University, Shanghai, China) were cultured in Dulbecco’s modified Eagle’s medium (DMEM, Invitrogen, Carlsbad, CA) containing 10% fetal bovine serum (FBS) in a humidified incubator at 37°C with an atmosphere of 5% CO_2_. HUVECs were purchased from Allcells (Shanghai, China) and cultured in Endothelial Cell Growth Medium-2 (ECM, Lonza, Basel, Switzerland ) with 10% FBS in a humidified incubator at 37°C with an atmosphere of 5% CO_2_.

### miRNA and Transfection

To modify miR-26a expression levels in HCC cell lines, we obtained recombinant lentivirus vectors from Genechem (Genechem, Shanghai, China) that included genes such as pre-miR-26a, the negative control precursor miRNA; anti-miRNA-Locked nucleic acids (LNAs) against miR-26a; and the negative control of anti-miRNA-LNAs. These vectors, with their packaging vectors, were transfected into 293T cells using Lipofectamine 2000 (Invitrogen, Carlsbad, CA). HepG2 cells or HCCLM3 cells were then transfected with virus following the manufacturer’s instruction, and Western blot and quantitative RT-PCR (qRT-PCR) were used to validate the transfection results.

### siRNA and transfection

Cells were transfected with either a non-specific control or an small interfering RNA (siRNA) (Genepharma, shanghai,China ) using Lipofectamine 2000 (invitrogen) in OPRI-MEM medium(Gibco) according to the manufacturer’s instructions and then incubated and used for further experiments. The sequence of siRNA was: VEGFA (5’-UUCUCCGAACGUGUCACGUTT-3’) and PIK3C2α (5’ -AAGGTTGGCACTTACAAGAAT-3’). 

### qRT-PCR for miR-26a

Cells were transfected with pre-miR-26a, miR-26a inhibitor, or their control oligomers. After 48 h, total RNA was extracted using TRIzol Reagent (Sigma, St. Louis, MO). Reverse transcription reactions were performed by using All-in-One miRNA qRT-PCR Detection Kit (GeneCopoeia, Rockville, MD). Briefly, the extracted RNA was reverse-transcribed in the presence of a poly-A polymerase with an oligo-dT adaptor. qPCR was then carried out using All-in-One miRNA qPCR primer (GeneCopoeia), according to the manufacturer’s instruction.

### Western Blot

Cells were lysed using cell lysis buffer (150 mM NaCl, 50 mM Tris-HCl, pH 8.0, 0.1% SDS, 1% Triton X-100) containing protease and phosphatase inhibitors. Equivalent amounts of whole cell extracts were subjected to SDS-PAGE gel and transferred to nitrocellulose membranes. The membranes were blocked with 5% non-fat milk for 2 h and then incubated with respective primary antibody overnight at 4°C, followed by the incubation of the appropriate HRP-conjugated secondary antibody for 2 h at room temperature. Blots were visualized with an ECL detection kit (Pierce, IL) and analyzed using Quantity One 1-D Analysis Software (Bio-Rad, San Francisco, CA).

### Collection of the Conditioned Medium (CM)

HCC cells were treated with 3-(5′-hydroxymethyl-2′-furyl)-1-benzylindazole (YC-1, 5 μM, Sigma-Aldrich, St. Louis, MO), LY294002 (20 μM, Beyotime, Jiangsu, China), , or vehicle for 12 h, and then incubated in DMEM with 0.1% BSA for 14 h followed by collection of the CM. The medium was spun down at 3000 rpm for 20 min, and the supernatant was collected and stored at −80°C. In the experiments of bevacizumab (Roche, Shanghai, China) blocking assay, bevacizumab and control IgG (final, 0.5 mg/ml) were added to CM 30 min before further experiments. 

### ELISA

VEGFA concentrations in the supernatants were measured by enzyme-linked immunosorbent assay (ELISA) kit (Boster, Wuhan, China) according to the manufacturer’s instruction. We collected the total cell protein to assess the different cell numbers of the different groups. An equal volume of lysis buffer was added before we extracted the total cellular protein, and then bicinchoninic acid (BCA) assay was used to measure the protein concentration. Thereafter, the VEGFA concentration was normalized to the total cellular protein.

### HUVEC Tube Formatting Assays

HUVECs (1.5 × 10^4^/well) were added to Matrigel-coated 96-well plates and incubated at 37°C for 6 h with various CMs. HUVECs were photographed under an inverted microscope. Tube formation was assessed by measuring the length of tube from 10 random fields at ×100 magnification. The relevant effect of CM was normalized to the total cellular protein.

### HUVEC Migration Assay

Quantitative cell migration assays were performed using a chamber (Corning, Tewksbury. MA) with 8.0-μm polycarbonate filter inserts in 24-well plates as described before [33]. Briefly, the lower chamber was filled with CM. HUVECs (5 × 10^4^ cells/well) in serum-free medium were added to the upper chamber. The cells were allowed to migrate for 12 h at 37°C. The non-migrated cells were removed from the upper surface of the membrane by scraping with a cotton swab, and the migrated cells were fixed with methanol, stained with crystal violet, and photographed under an inverted microscope. Migration was assessed by counting the number of stained cells from 10 random fields at ×100 magnification. The relevant effect of CM was normalized to the total cellular protein.

### Luciferase Activity Assay

HEK293T cells in a 96-well plate were transfected with 50 ng of miR-26a expression vector, control vector, or negative control. The cells were then co-transfected with 10 ng of vector with the wild-type (WT) or mutant 3′UTR of the target gene. Forty-eight hours later, renilla luciferase activity was measured using a dual-luciferase reporter system (Promega, Madison, WI) and detected using an Orion II microplate luminometer (Berthold, Bad Wildbad, Germany). Luciferase reporter assays were performed in quadruplicate and repeated in three independent experiments. 

### Xenograft Model of HCC in Nude Mice

Male BALB/c nude mice (5 weeks old) were purchased from the Shanghai Institute of Materia Medica, Chinese Academy of Science, and housed under specific pathogen-free conditions. The experimental protocol was approved by the Shanghai Medical Experimental Animal Care Commission. Twenty-four mice were randomized into four groups and various cells (6 × 10^6^ cells) in 200 μl normal saline were implanted by subcutaneous injection to obtain subcutaneous tumors. The tumor volumes were measured by vernier caliper every 4 days, and the mice were euthanized after 4 weeks. The xenograft HCC model was established by orthotopic implantation of histologically intake tumor tissue into the nude mouse liver [34]. The mouse body weights were measured by electronic balance every week, and the mice were euthanized after 5 weeks. After measured the tumor volume, the tumors were removed from liver into 4% paraformaldehyde solution. The tumor volume was calculated according to the formula: tumor volume = (largest diameter × perpendicular height^2^)/2.

### Immunohistochemical (IHC) Analysis

Paraffin-embedded tumor tissues were cut into standard 6-μm sections. IHC of VEGFA (1:100; Santa Cruz, Santa Cruz, CA) and CD31 (1:100; Abcam, Cambridge, MA) was performed in the sections on slides [33]. The integrated optical density (for VEGFA) or area (for CD31) of positive staining/total area was quantified by Image-Pro Plus software (Media Cybernetics Inc, Bethesda, MD) [35].

### Patients and Follow-Up

HCC specimens used in tissue microarray analysis were obtained from our previous study [26] and obtained with informed consent from patients who underwent radical resection between 1999 and 2003 at the Liver Cancer institute and Zhongshan Hospital (Fudan University, Shanghai, China). None of the patients received any preoperative anticancer treatment. The research was approved by the research ethics committee of Zhongshan Hospital. A total of 102 cases were used to examine VEGFA expression, and 73 cases had the miR-26a expression data we detected in previously study. The patients’ clinicopathologic features are detailed in supplementary Table. All the patients were followed up until 2011 with a median observation time of 60.8 months. This study was approved by The Zhongshan Hospital Research Ethics Committee. Informed consent was obtained from each patient according to the committee’s regulation. Participants provided their written informed consent to participate in this study. 

### Tissue Microarrays and Immunohistochemistry

As previously described [36], tissue microarrays were constructed and immunostaining was done using a two-step protocol as previously described. The measurement of VEGFA expression used a computerized image system composed of a Leica CCD camera DFC420 connected to a Leica DM IRE2 microscope (Leica Microsystems Imaging Solutions Ltd, Cambridge, UK). The photographs of four representative fields were captured by Leica QWin Plus v3 software, and the integrated optical density values of those photographs were measured by Image-Pro Plus v6.0 software. For the reading of each antibody staining, a uniform setting for all the slides was applied. 

### Statistical Analysis

Analysis was done using SPSS 18.0 for Windows (SPSS, Inc.). Quantitative variables were analyzed using the *t* test or the ANOVA test or Spearman correlation test. *P* < 0.05 was considered statistically significant.

## Results

### miR-26a Inhibited the Expression of VEGFA in HCC Cells

**Figure 1:**
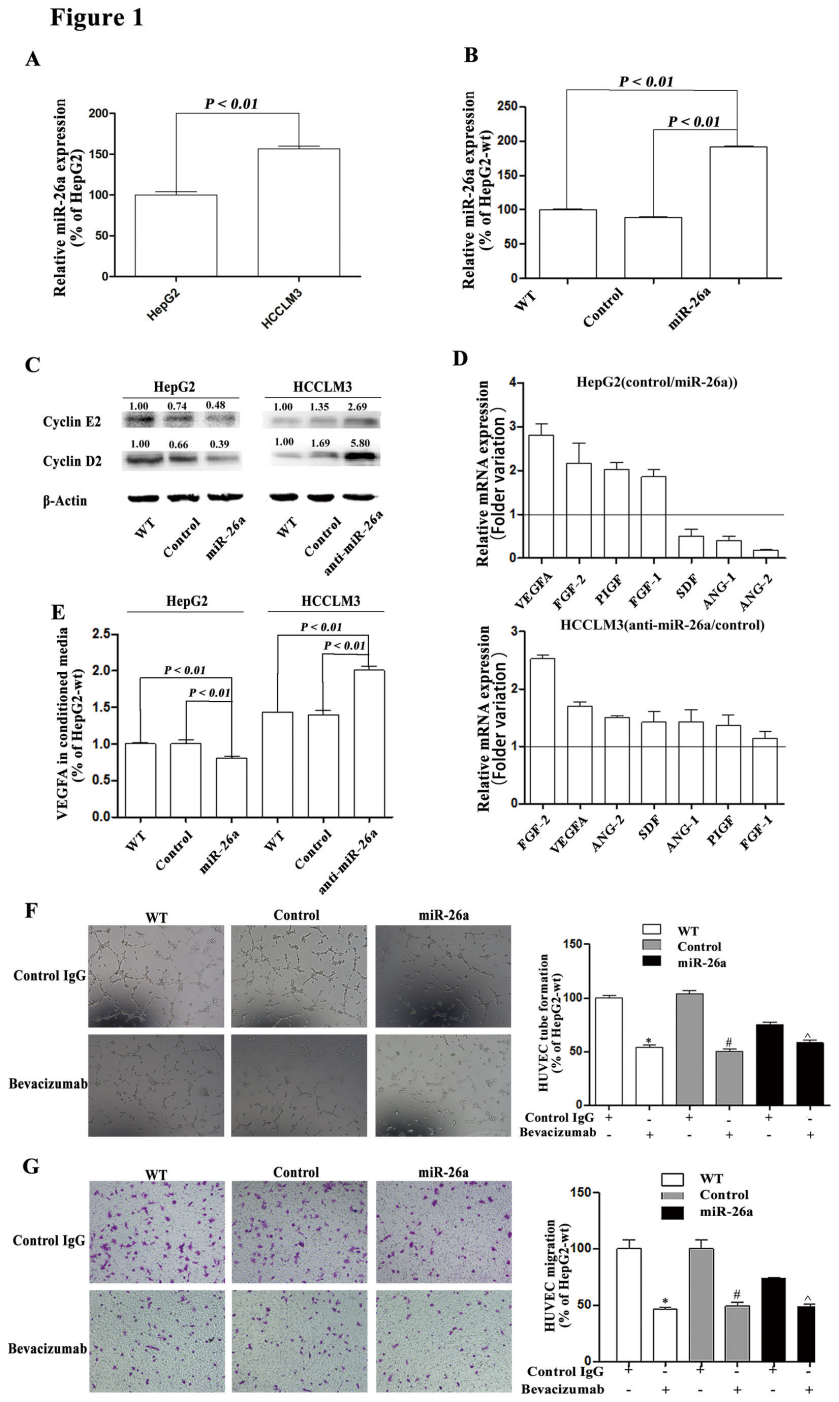
Modifications of miR-26a in HCC cell lines and detection of Akt/HIF-1ɑ/VEGFA pathway expressions. (A) miR-26a expression in HCCLM3 and HepG2 cells was detected by qRT-PCR. (B) miR-26a expression level in HepG2 cells after transfection with miR-26a was assessed by qRT-PCR. (C) The expression of Cyclin D2 and Cyclin E2, two validated targets of miR-26a, in all HCC cells after transfection. (D) The relative mRNA levels of seven pro-angiogenic factors (VEGFA, FGF-1, FGF-2, ANG-1, ANG-2, PIGF, and SDF) were detected by qRT-PCR. (E) The section level of VEGFA in the CM from all HCC cells was assessed by ELISA. (F) HUVECs were treated with CM from all HepG2 cells with addition of bevacizumab or control IgG, and tube formation and migration were assessed. The tube length and the number of migrating cells were evaluated by counting 10 random fields at ×100 magnification. * compare to HepG2-wt, *P* < 0.05; # compare to HepG2-control, *P* < 0.05; ^ compare to HepG2-miR-26a, *P* < 0.05.

The miR-26a expression in HCCLM3 cells was higher than that in HepG2 cells (Figure 1A). After modifying miR-26a expression levels in HepG2 and HCCLM3 cells, we established subclones with stable expression of miR-26a. We named those cells HepG2-wt (wild type of HepG2), HepG2-control (HepG2 transfected with the negative control of precursor miRNA), and HepG2-miR-26a (HepG2 transfected with pre-miR-26a), or HCCLM3-wt (wild type of HCCLM3), HCCLM3-control (HCCLM3 transfected with the negative control of anti-miRNA-LNAs), and HCCLM3-anti-miR-26a (HCCLM3 transfected with anti-miRNA-LNAs against miR-26a). The expression of miR-26a was elevated in HepG2-miR-26a cells after transfection (Figure 1B). Expression of cyclin D2 and E2, the target genes of miR-26a [28], was also down-regulated in HepG2-miR-26a cells and up-regulated in HCCLM3-anti-miR-26a cells after transfection (Figure 1C). 

To explore whether miR-26a affects angiogenesis, we treated HUVECs with the CM from HCC cells. We found CM from HepG2-miR-26a cells inhibited tube forming and migration of HUVECs, whereas CM from HCCLM3-anti-miR-26a cells promoted tube forming and migration of HUVECs, when compared with CM from their wild-type cells (Figure S1). 

To explore which angiogenesis-related cytokine were affected by miR-26a, we examined mRNA expression of VEGFA, FGF-1, FGF-2, Ang-1, Ang-2, PIGF, and SDF-1, and found VEGFA expression decreased significantly in HepG2-miR-26a cells but increased significantly in HCCLM3-anti-miR-26a cells (Figure 1D). We then measured protein concentration of VEGFA in CM and found up-regulation of miR-26a significantly decreased the protein level of VEGFA and down-regulation of miR-26a increased the protein level of VEGFA in CM (Figure 1E). Furthermore, inhibition of VEGFA by bevacizumab and VEGFA siRNA approach was used to validate the relation between VEGFA and miR-26a expression. Both bevacizumab and VEGFA siRNA treatment eliminated the difference of effect on HUVECs among CMs from HepG2 -wt, HepG2-control, and HepG2-miR-26a cells (Figure 1F, 1G and Figure S2). Similar results were also found in HCCLM3 cells (Figure S3),

### miR-26a Affected VEGFA Expression through the PI3K/Akt/HIF-1α/VEGFA Pathway

**Figure 2:**
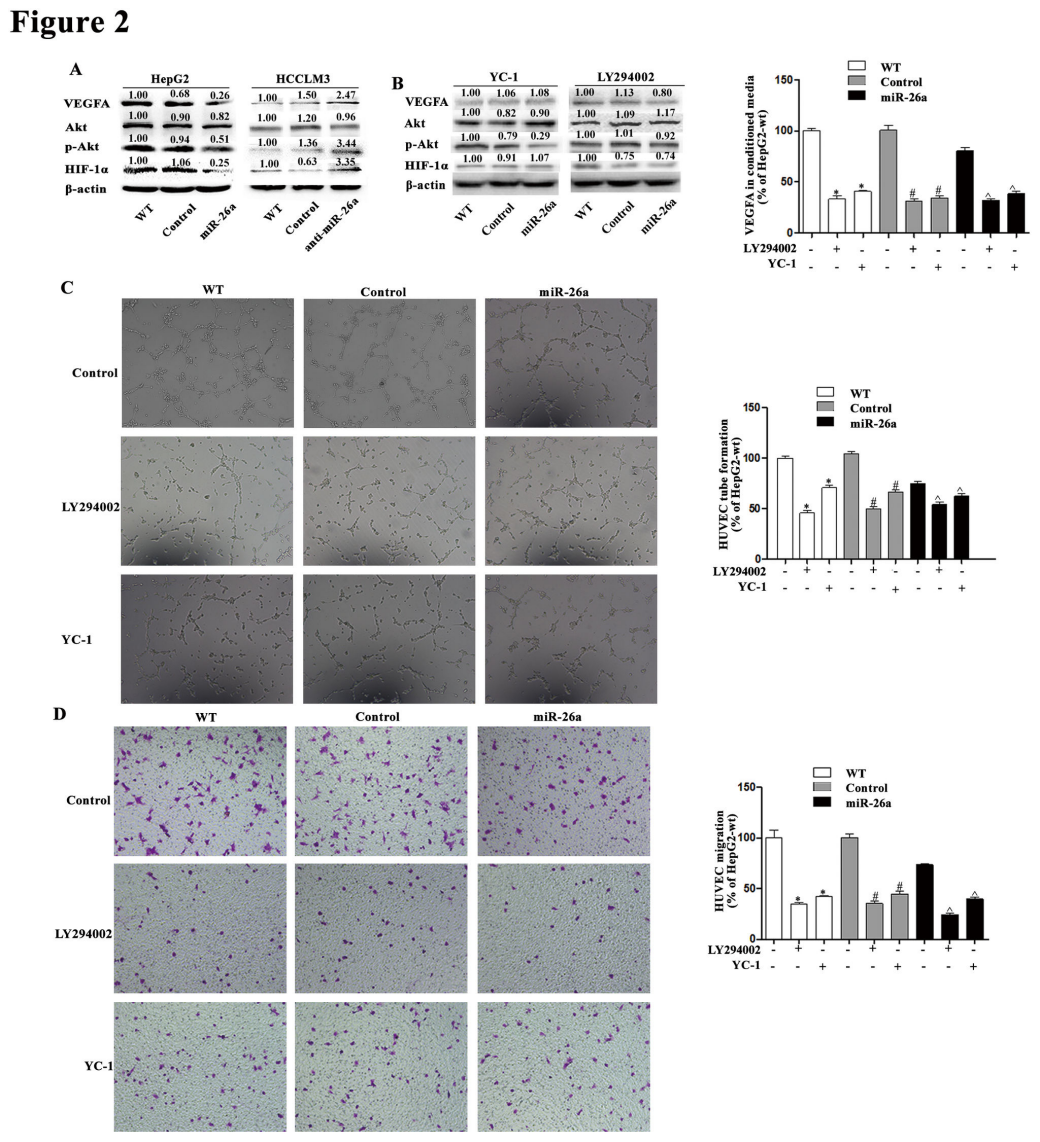
The anti-angiogenic effect of miR-26a is mainly though regulation of the PI3K/Akt/HIF/VEGFA pathway in HepG2 cells. HIF-1α inhibitor, YC-1, PI3K inhibitor, and LY294002 were used to treat HCC cells. (A) The expression of VEGFA, Akt, p-Akt, and HIF-1α in all HCC cells. (B) After YC-1 or LY294002 was used to treat HepG2 cells with or without transfection of miR-26a, the expression of VEGFA was assessed by Western blot and ELISA. (C, D) After YC-1 or LY294002 was used to treat HepG2 cells, CM was collected and the effect of CM on HUVEC tube formation and migration was assessed. The tube length and the number of migrating cells were evaluated by counting 10 random fields at ×100 magnification. * compare to HepG2-wt, *P* < 0.05; # compare to HepG2-control, *P* < 0.05; ^ compare to HepG2-miR-26a, *P* < 0.05.

Because HIF-1α is an important inducer of VEGFA [37–40] and the PI3K/Akt pathway regulates VEGFA and HIF-1α expression [11,33], we further measured the expression of Akt, phosphorylated Akt (p-Akt), and HIF-1α in these cells. The results showed expression of p-Akt and HIF-1α was decreased in HepG2-miR-26a cells and increased in HCCLM3-anti-miR-26a cells, whereas the expression of total Akt was not affected by miR-26a (Figure 2A).

To determine whether PI3K/Akt/ HIF-1α pathway was involved in this process, PI3K inhibitor LY294002 and HIF-1α inhibitor YC-1 were used (Figure 2B). HUVEC tube formation and migration were significantly inhibited by the CM from HepG2 cells treated with LY294002 and YC-1. In addition, LY294002 and YC-1 eliminated the difference in pro-HUVEC effect of CM among HepG2 -wt, HepG2-control, and HepG2-miR-26a cells (Figure 2C and 2D). Similar results were also found in HCCLM3 cells (Figure S4).

### PIK3C2α Is a Novel Target of miR-26a

**Figure 3:**
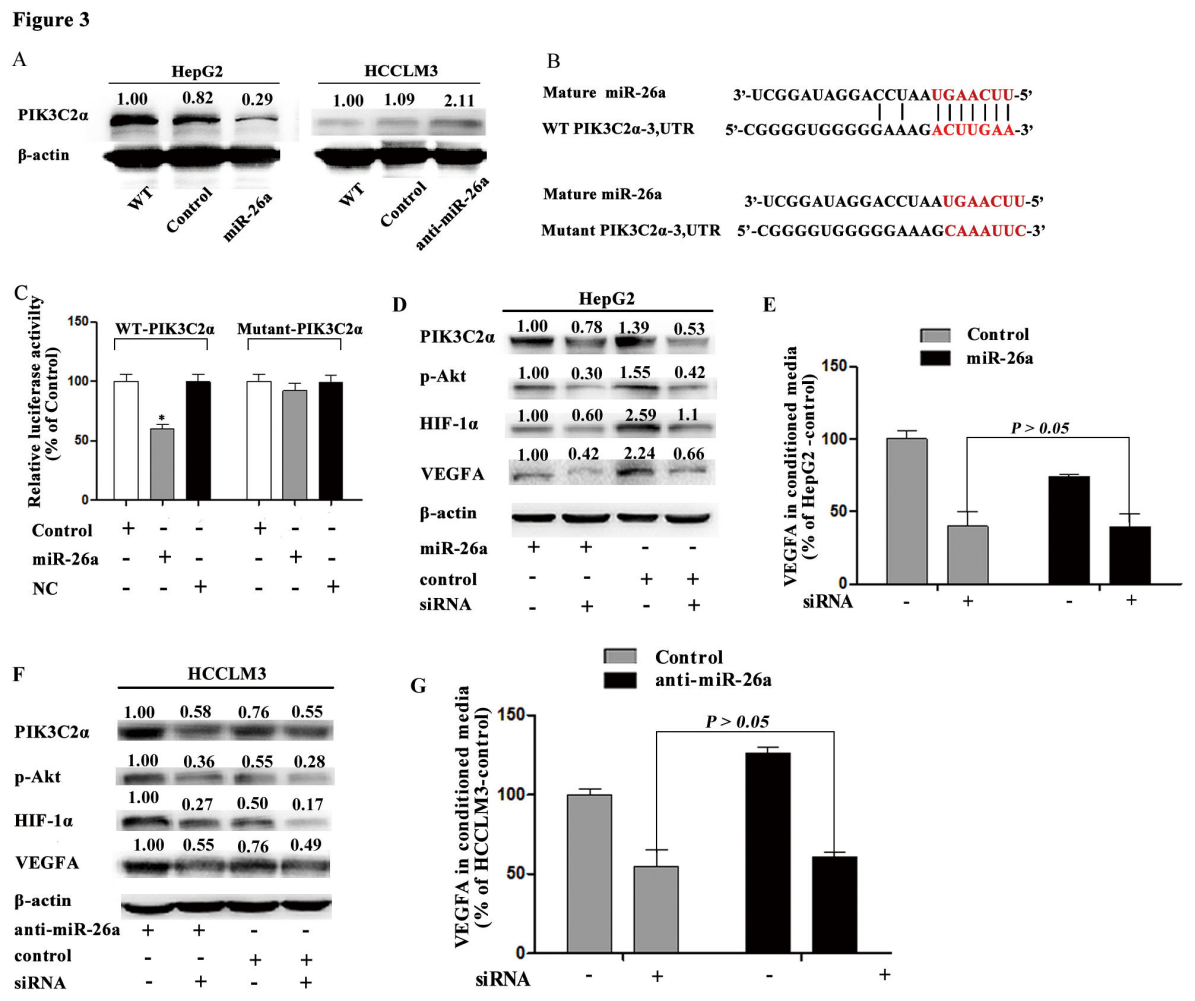
PIK3C2α is a direct target of miR-26a in HCC cells. (A) The expression of PIK3C2α in HCC cells was examined by Western blot. (B) The putative binding sequence of miR-26a in the 3′-UTR of PIK3C2α is shown. (C) Luciferase activity assay was used to detect the role of miR-26a on the 3′-UTR of PIK3C2α. NC: the mock of precursor miRNA, * compare to the control of Luciferase activity assay, *P* < 0.05. (D, E) After PIK3C2α siRNA was used to transfect HepG2 cells with or without transfection of miR-26a, the expression of VEGFA was assessed by Western blot and ELISA. (F, G) After PIK3C2α siRNA was used to treat HCCLM3 cells with or without transfection of anti-miR-26a, the expression of VEGFA was assessed by Western blot and ELISA.

We have showed miR-26a decreased VEGFA expression, probably via PI3K/Akt/HIF-1α/VEGFA, we explored which molecule is the direct target of miR-26a in this pathway. Using targetscan (www.targetscan.org) [41], PIK3C2α was identified as a possible target of miR-26a. Moreover, we found that the expression of PIK3C2α was decreased after transfection with pre-miR-26a in HepG2 cells and increased after down-regulating miR-26a in HCCLM3 cells (Figure 3A). A luciferase activity assay was used to confirm that miR-26a could directly regulate PIK3C2α expression. The 3′UTR of PIK3C2α mRNA, containing a miR-26a putative binding site or the mutant form in which the putative miR-26a binding site can be mutated, was cloned downstream of the luciferase reporter gene (Figure 3B). We found miR-26a interacted with PIK3C2α mRNA and inhibited PIK3C2α protein expression (Figure 3C).

Because PIK3C2α belongs to class II PI3Ks[42], and PI3K/Akt pathway can regulated VEGF and HIF-1ɑ expression[11,33], we transfected all of the tested cell lines with PIK3C2α siRNA to confirm whether miR-26a regulated VEGFA expression by inhibiting PIK3C2α expression. We found PIK3C2α siRNA reduced VEGFA expression or secretion and eliminated the difference of those between HepG2-control and HepG2-miR-26a cells (Figure 3D), and between HCCLM3-control and HCCLM3-anti-miR-26a cells (Figure 3E).

### miR-26a Inhibited Tumor Growth and Angiogenesis in HCC Xenograft Models

**Figure 4:**
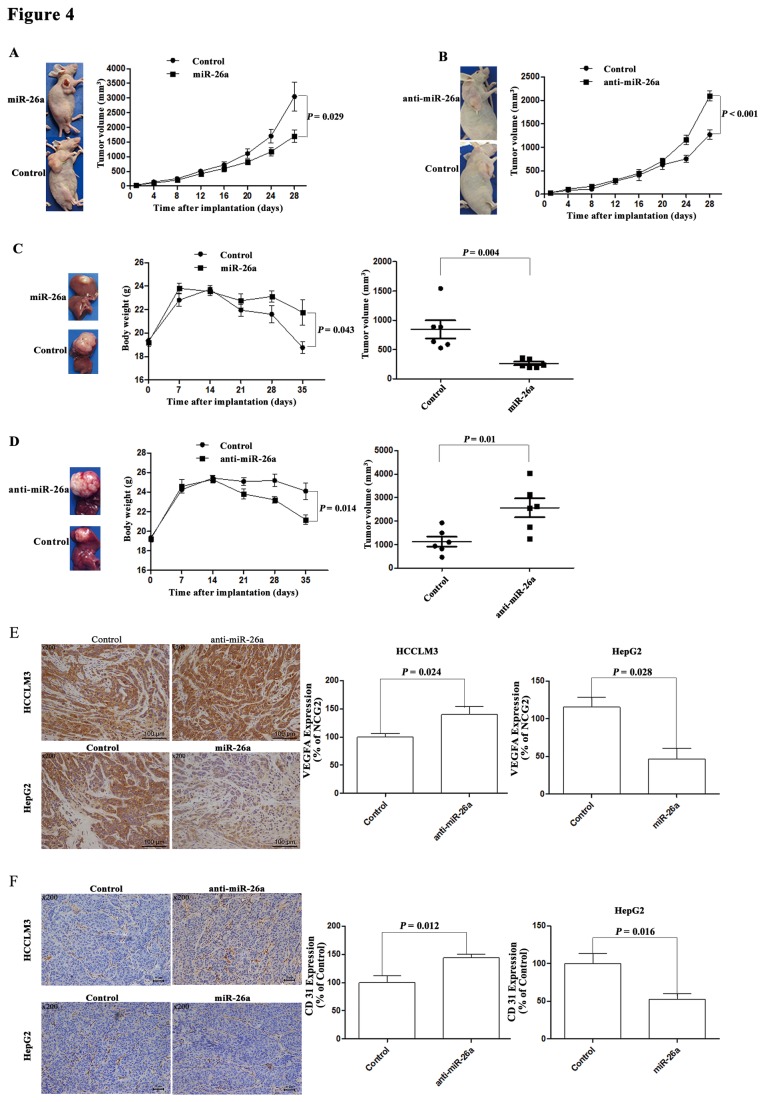
The role of miR-26a on tumor angiogenesis in vivo. (A, B) HepG2-control, HepG2-miR-26a, HCCLM3-control, and HCCLM3-anti-miR-26a (6 × 10^6^) were injected into the right flanks of the nude mice, and the tumor growth curves of HepG2 (A) and HCCLM3 (B) were assessed. (C, D) HCC subcutaneous tumor tissues were implanted into nude mouse livers to establish the xenograft HCC model. After 35 days, the mice were euthanized and their body weights and tumor volumes were assessed. (E, F) HCC tumor tissues from the orthotropic implantation model were used to stain for VEGFA (E) and CD31 (F) through immunohistochemistry. Representative images are shown (200×).

To evaluate the role of miR-26a in tumor growth, we examined its effects in HepG2 and HCCLM3 subcutaneous and orthotopic models. We found the HepG2-miR-26a tumors had a decreased volume in comparison with HepG2-control tumors (*P* = 0.029; Figure 4A), and the HCCLM3-anti-miR-26a tumors showed an increased volume in comparison with its HCCLM3-control tumors (*P* < 0.001; Figure 4B). In the orthotopic models, mice with HepG2-miR-26a tumors had a higher body weight and smaller tumor size than those with HepG2- control tumors (*P* = 0.043 and *P* = 0.004; Figure 4C). Moreover, mice expressing HCCLM3-anti-miR-26a tumors had lower body weights and larger tumor sizes than the mice with HCCLM3-control tumors ((*P* = 0.014 and *P* = 0.01; Figure 4D). 

We further examined VEGFA expression and microvessel density (MVD) in the tumor samples. HepG2-miR-26a tumors had lower VEGFA expression and lower MVD compared with the HepG2-control tumors (*P* = 0.0028 and *P* = 0.016; Figure 4E). HCCLM3-anti-miR-26a tumors had higher VEGFA expression and MVD than that of the HCCLM3-control tumors (*P* = 0.0024 and *P* = 0.012; Figure 4E).

### miR-26a Expression Was Inversely Correlated with VEGFA Expression in Tumor Specimens from Patients

**Figure 5:**
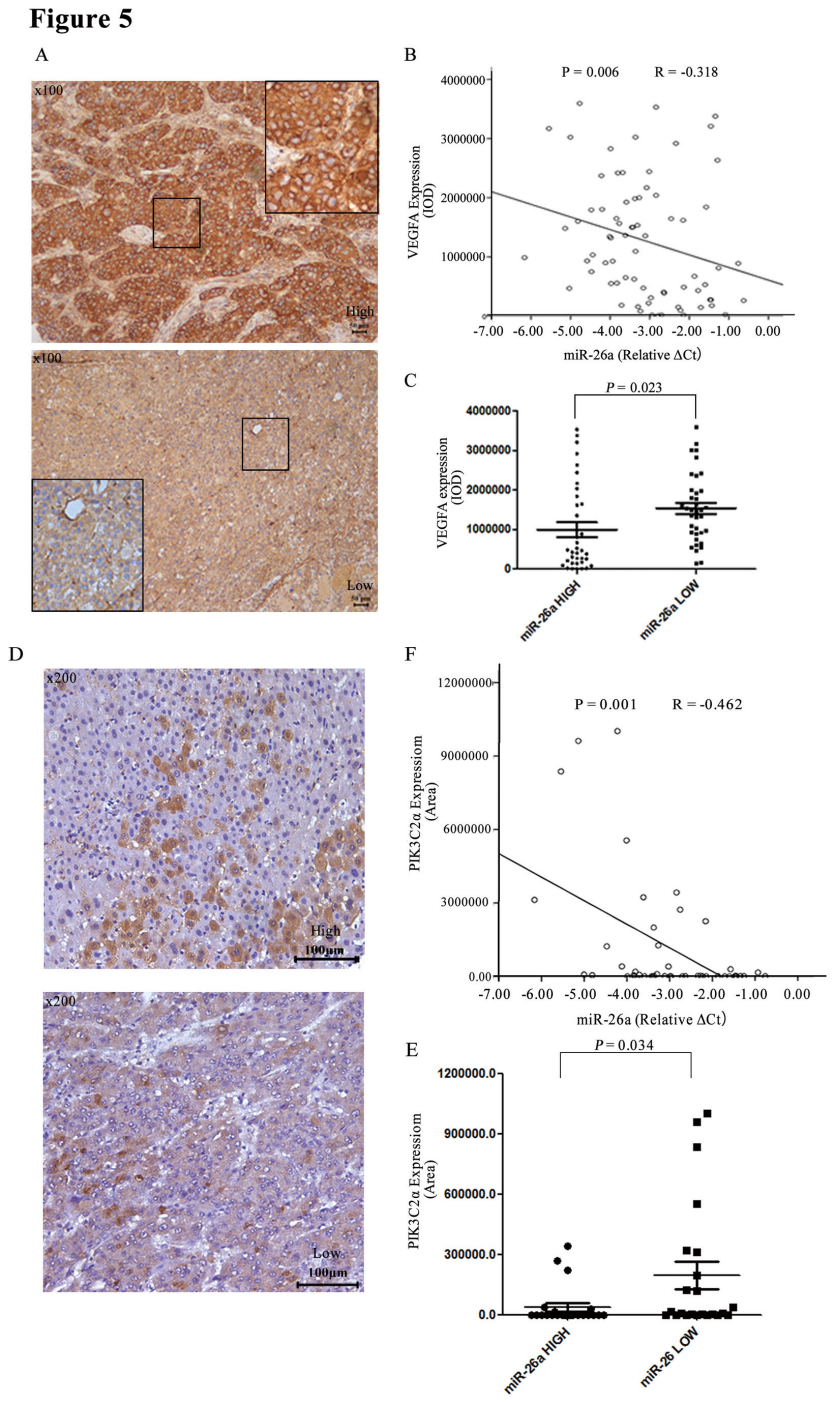
Expression of VEGFA in HCC tissue and the relationship between VEGFA and miR-26a. (A) The HCC tissue microarray containing 102 cases was used to analyze VEGFA expression through immunohistochemistry. Representative images are shown (100×). (B, C) A total of 73 cases from among the cases composing the tissue microarray were used to analyze the relationship between miR-26a and VEGFA. (D) The HCC tissue microarray containing 47 cases was used to analyze PIK3C2α expression through immunohistochemistry. Representative images are shown (200×). (E, F) The data of immunohistochemistry were used to analyze the relationship between miR-26a and and PIK3C2α expression.

We examined the relationship between miR-26a and VEGFA in 102 HCC patients. The data on MiR-26a expression for the patients were obtained from our previous study, and the representative images of the immunostaining for VEGFA are shown in Figure 5A. There was an inverse correlation between miR-26a and VEGFA expression (*P* = 0.006, *R* = −0.318; Fig. 5B). Using a cutoff point at the median value for miR-26a expression, we divided the patients into two groups and found that patients with the higher miR-26a expression had lower VEGFA expression compared with those with low miR-26a expression (*P* = 0.023; Figure 5C). These results further support a relationship between miR-26a and VEGFA expression. Similar results were also found in the analysis of relationship between miR-26a and PIK3C2α in 47 patients, who belong to the cohort we have studied and published previously [26] (Figure 5A, 5D and 5E). 

## Discussion

Here we demonstrated that miR-26a is associated with decreased tumor angiogenesis in HCC, at least partly through decreasing VEGFA expression by directly inhibiting PIK3C2α. miRNAs have been reported to be involved in regulation of angiogenesis through different mechanisms, including miR-214 reducing the secretion of the hepatoma-derived growth factor in human hepatoma [43], miR-221 regulating andiogenin and CXCL16 expression levels [44], and miR-29b reducing the expression of matrix metalloproteinase 2 [45]. This is the first report to show that miR-26a can inhibit angiogenesis by regulating VEGFA production in HCC.

Our previous studies indicated that miR-26a expression was associated with HCC, and low miR-26a expression indicated a shorter overall survival time in HCC patients [26]. However, the mechanisms were not fully illustrated. Kota et al. reported that miR-26a can inhibit cancer cell proliferation in an HCC mouse model [28], and later studies reported that miR-26 inhibited the G1/S transition by activating the pRb protein [29] and miR-26a suppressed tumor growth by the IL-6-stat3 signaling pathway [31]. The present study uncovered a novel target of miR-26a and revealed its role in tumor angiogenesis in HCC.

HIF-1α is a transcription activator of the VEGFA promoter, and it can be induced by hypoxia or the activation of ERBB2, SRC, endothelin-1, the RAS/MAPK pathway, and the phosphoinositide 3-kinase (PI3K)-Akt-mTOR pathway [33]. The PI3K-Akt-HIF-1α-VEGFA pathway plays an important role in tumor angiogenesis. PI3Ks comprise a family of enzymes that phosphorylate membrane inositol lipids at the 3′ position of the inositol ring. The lipid products of PI3Ks serve as important intracellular messengers by interacting with effector proteins, including protein kinases, guanine nucleotide exchangers for G proteins, and actin cytoskeleton-regulating proteins[17]. Through these actions, PI3Ks regulate a diverse array of cellular processes. It has been reported that some miRNAs can regulate tumorigenesis by targeting PI3Ks. Fang et al. reported that miR-7 can inhibit HCC tumor growth and metastasis by targeting PIK3CD [46]. Zhu et al. found that miR-126 increases the sensitivity of NSCLC cells to anticancer agents through negative regulation of a VEGF/PI3K/Akt/MRP1 pathway [47]. Zhu et al. also reported that miR-126 can affect tumor genesis and growth by targeting PI3KR2 and regulating the VEGF/PI3KR2/Akt pathway in human breast cancer [48]. But so far, there has been no report on whether miRNA can regulate VEGFA expression by targeting PI3KC2α. PIK3C2α is a class II PI3K and has an essential role in angiogenesis [49]. It can regulate the Akt pathway and VEGFA-induced tube formation or transwell migration [17,41]. Our present study found PIK3C2α is a new target of miR-26a, and miR-26a inhibited HCC angiogenesis mainly through suppressing the PIK3C2α/p-Akt/HIF-1α/VEGFA pathway. 

This study has some potential limitations. Our result showed miR-26a may inhibit angiogenesis through the PIK3C2α/Akt/HIF-1α/VEGFA Pathway. However, the regulation of angiogenesis-related cytokines in cancer cell is a very complicated network, we did not exclude other signal pathways which may modulate VEGFA expression and can be affected by miR-26a. 

It was recently reported that some molecules or cells played an important role in HCC progression and might be the potential target for HCC therapy, such as Polo-like kinase 1, the insulin-like growth factor and cytokine-induced kill cells[50–52]. Our findings present evidence that miR-26a suppresses HCC progression by modulating angiogenesis through the PIK3C2α/Akt/HIF-1α/VEGFA pathway. And the fundamental role of miR-26a in the angiogenesis of HCC also suggests that it could be a potential therapeutic target and provide a new basis for targeted molecular therapy of HCC.

## Supporting Information

Figure S1 **Effect of miR-26a on the tube formation and migration of HUVECs.** After the HCC cells were transfected with miR-26a or anti-miR-26a inhibitor or their negative control, the CM was collected and the effects of CM on HUVEC tube formation (A) and migration (B) were assessed. The tube length and the number of migration cells were evaluated by counting 10 random fields at ×100 magnification.
(TIF)

Figure S2 **miR-26a affected angiogenesis by regulating VEGFA expression in HepG2 cells.** (A, B) After the HepG2 cells were transfected with VEGFA siRNA, the expression of VEGFA was assessed by Western blot and ELISA. (C, D) After VEGFA siRNA was used to treat HepG2 cells, CM was collected and the effect of CM on HUVEC tube formation and migration was assessed. The tube length and the number of migrating cells were evaluated by counting 10 random fields at ×100 magnification.
(TIF)

Figure S3 **miR-26a affected angiogenesis by regulating VEGFA expression in HCCLM3 cells.** (A, B) HUVECs were treated with CM from all HCCLM3 cells with addition of bevacizumab or control IgG, and the tube formation and migration were assessed. (C, D) After the HCCLM3 cells were transfected with VEGFA siRNA, the expression of VEGFA was assessed by Western blot and ELISA. (E, F) After VEGFA siRNA was used to treat HCCLM3 cells, CM was collected and the effect of CM on HUVEC tube formation and migration was assessed. The tube length and the number of migrating cells were evaluated by counting 10 random fields at ×100 magnification.
(TIF)

Figure S4 **The anti-angiogenic effect of miR-26a was mainly mediated by PI3K/Akt/HIF/VEGFA pathway in HCCLM3 cells.** (C) After YC-1 or LY294002 was used to treat HCCLM3 cells with or without transfection of anti-miR-26a inhibitor, the expression of VEGFA was assessed by Western blot and ELISA. (D, E) After YC-1 or LY294002 was used to treat HCCLM3 cells, CM was collected and the effects of CM on HUVEC tube formation and migration were assessed. The tube length and the number of migration cells were evaluated by counting 10 random fields at ×100 magnification. * compare to WT-HCCLM3, *P* < 0.05; # compare to Control-HCCLM3, *P* < 0.05; ^ compare to anti-miR-26a-HCCLM3, *P* < 0.05.
(TIF)

Table S1 **A total of 102 patients who underwent curative liver resection for pathology-proven HCC in our institute were enrolled.** None of them received any preoperative anticancer treatment. These patients were observed between 1999 and 2003, and the clinicopathological features of them were shown.
(DOC)
