# Supplementary figures and images for: MicroRNA-26a Inhibits Angiogenesis by Down-Regulating VEGFA through the PIK3C2α/Akt/HIF-1α Pathway in Hepatocellular Carcinoma
Source: PLoS One. 2013 Oct 23;8(10):e77957. doi: 10.1371/journal.pone.0077957 (PMC3806796; doi:10.1371/journal.pone.0077957)

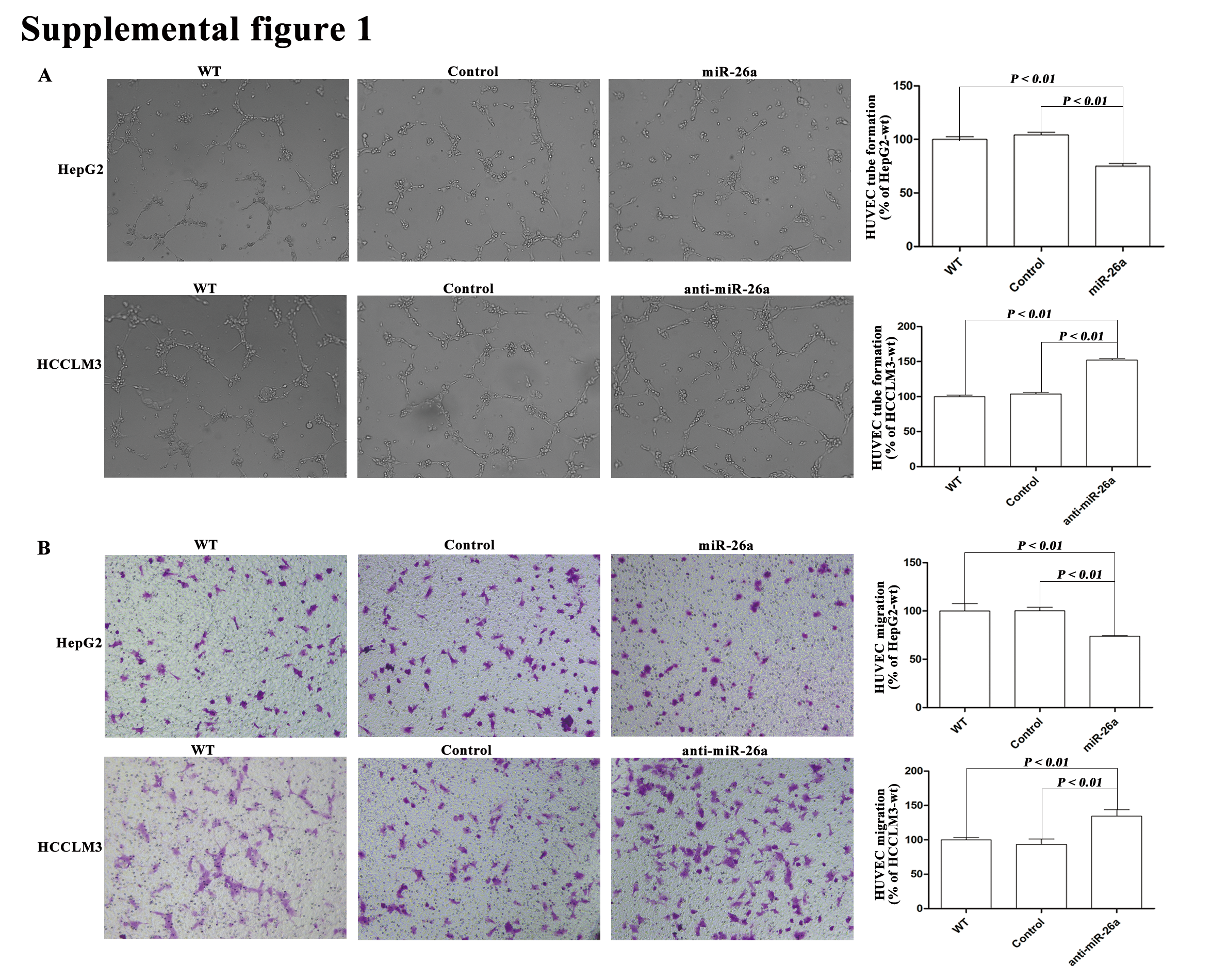

Supplement: Figure S1 — Effect of miR-26a on the tube formation and migration of HUVECs. After the HCC cells were transfected with miR-26a or anti-miR-26a inhibitor or their negative control, the CM was collected and the effects of CM on HUVEC tube formation (A) and migration (B) were assessed. The tube length and the number of migration cells were evaluated by counting 10 random fields at ×100 magnification. (TIF) [file pone.0077957.s001.tif]

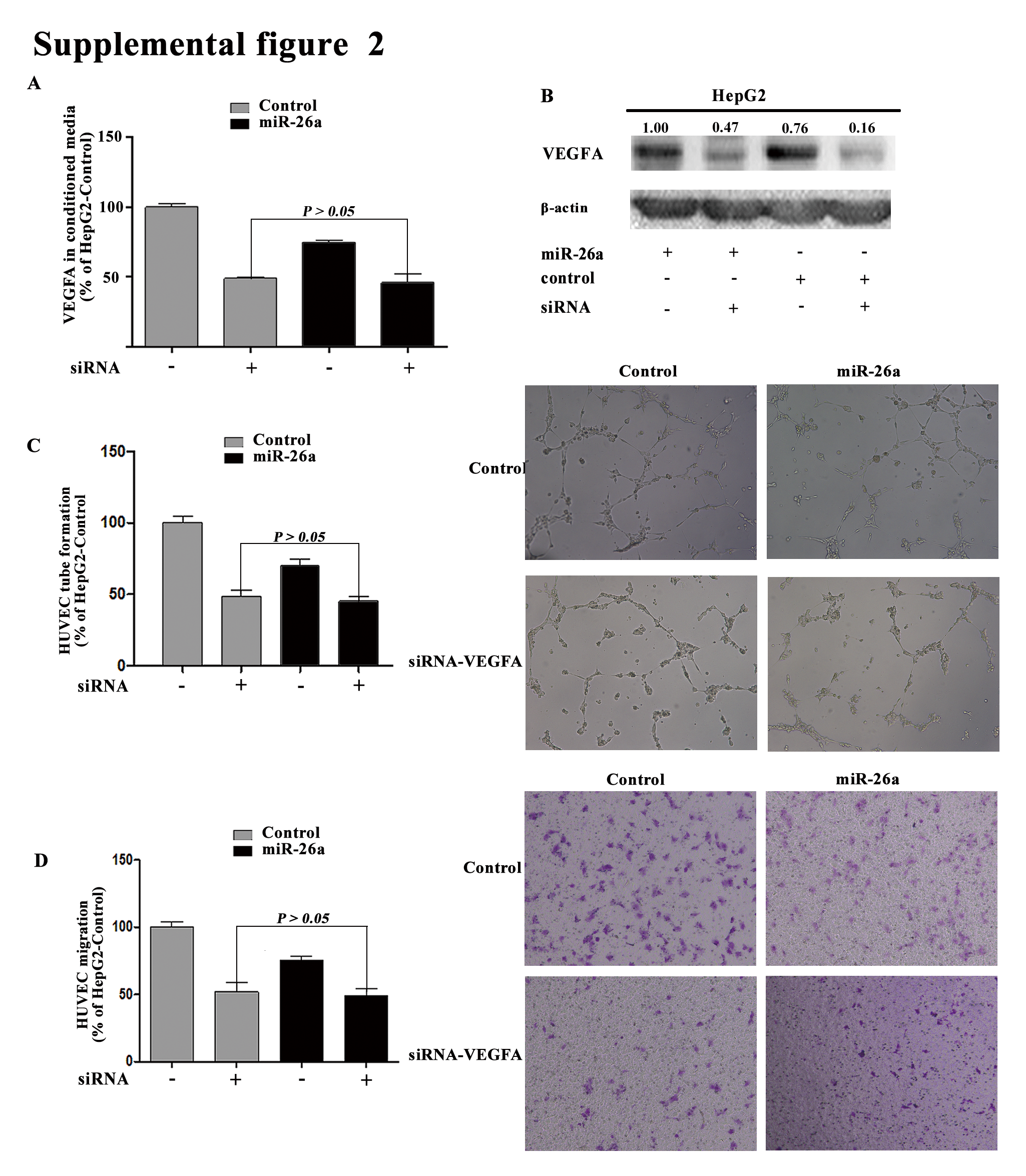

Supplement: Figure S2 — miR-26a affected angiogenesis by regulating VEGFA expression in HepG2 cells. (A, B) After the HepG2 cells were transfected with VEGFA siRNA, the expression of VEGFA was assessed by Western blot and ELISA. (C, D) After VEGFA siRNA was used to treat HepG2 cells, CM was collected and the effect of CM on HUVEC tube formation and migration was assessed. The tube length and the number of migrating cells were evaluated by counting 10 random fields at ×100 magnification. (TIF) [file pone.0077957.s002.tif]

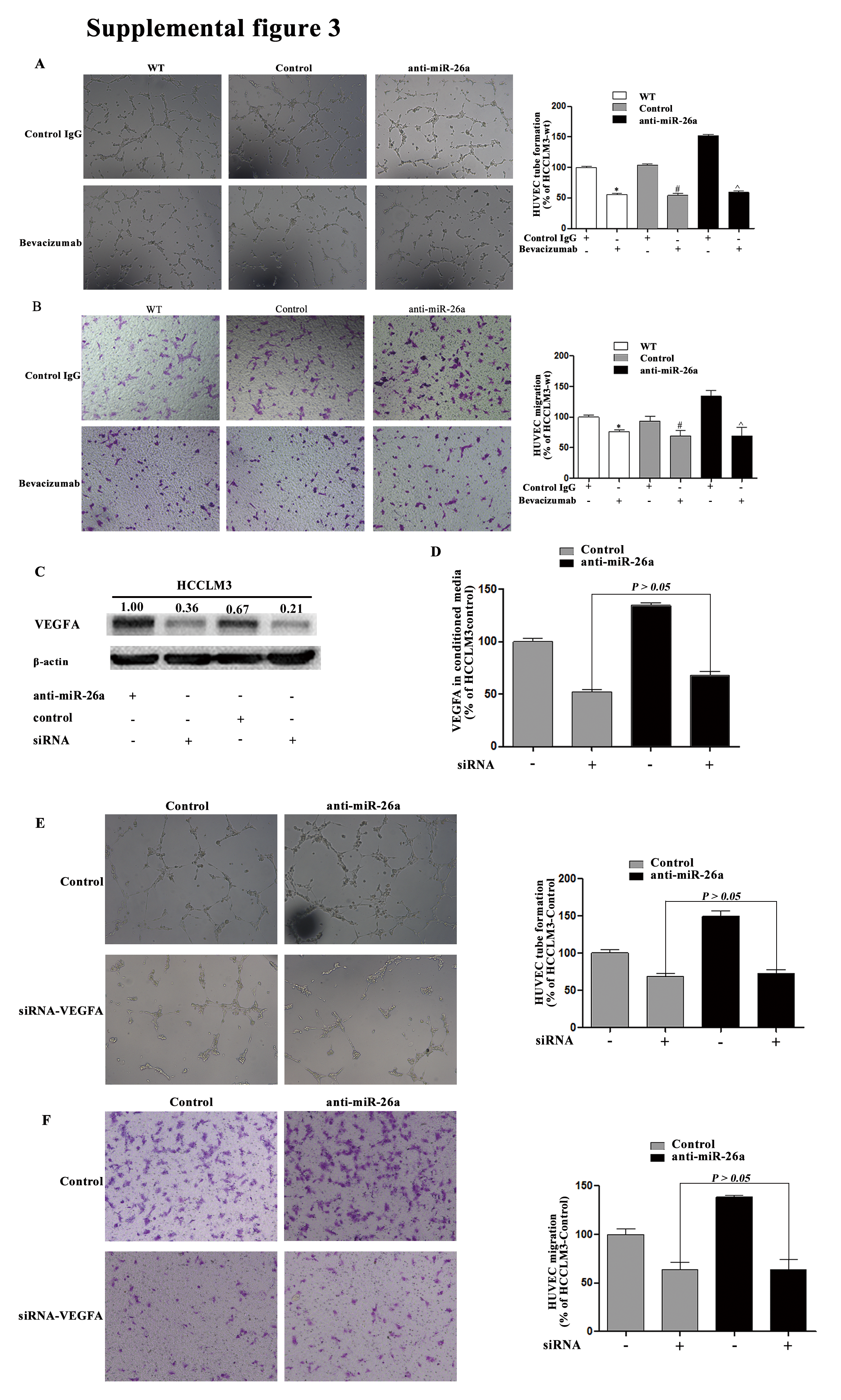

Supplement: Figure S3 — miR-26a affected angiogenesis by regulating VEGFA expression in HCCLM3 cells. (A, B) HUVECs were treated with CM from all HCCLM3 cells with addition of bevacizumab or control IgG, and the tube formation and migration were assessed. (C, D) After the HCCLM3 cells were transfected with VEGFA siRNA, the expression of VEGFA was assessed by Western blot and ELISA. (E, F) After VEGFA siRNA was used to treat HCCLM3 cells, CM was collected and the effect of CM on HUVEC tube formation and migration was assessed. The tube length and the number of migrating cells were evaluated by counting 10 random fields at ×100 magnification. (TIF) [file pone.0077957.s003.tif]

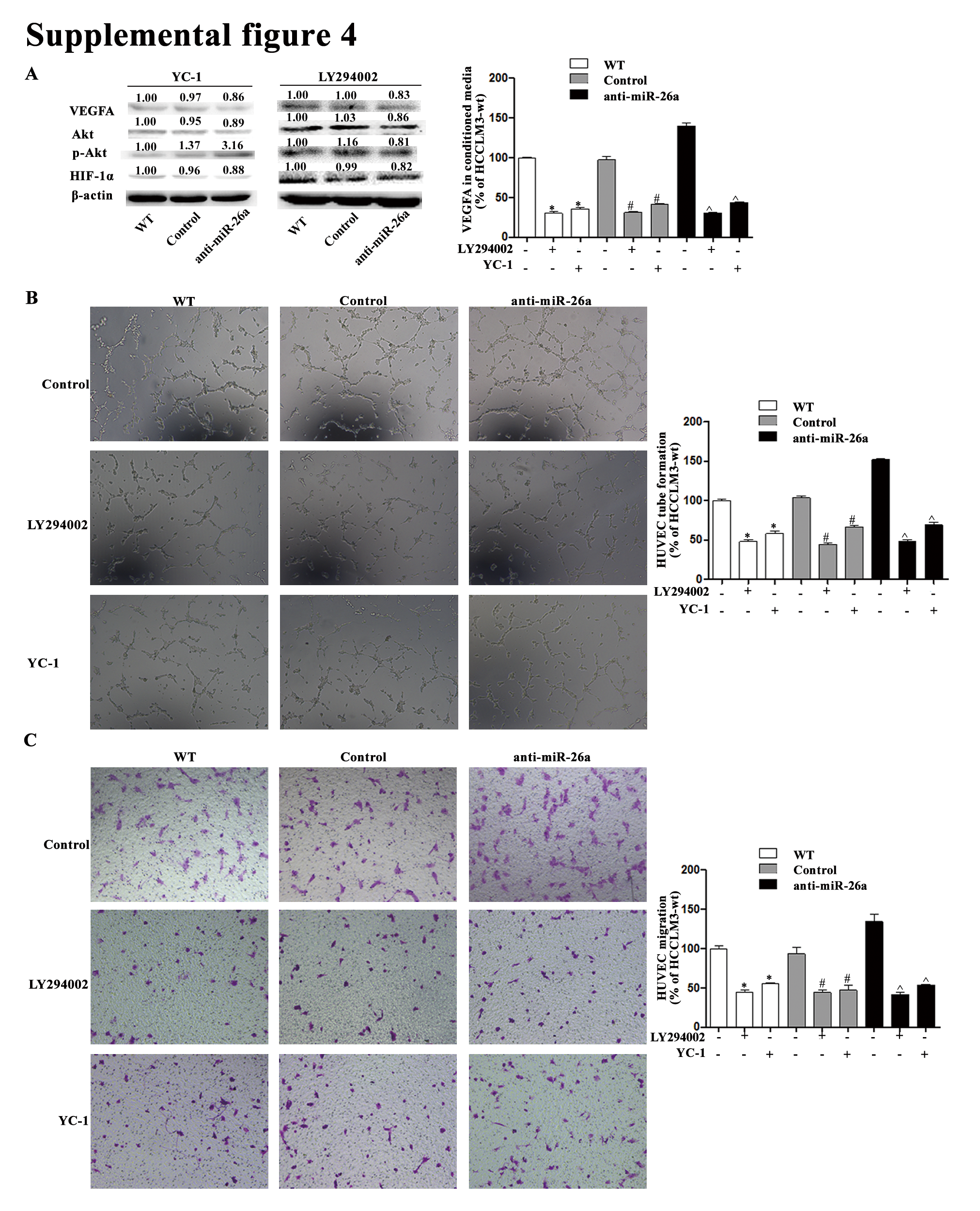

Supplement: Figure S4 — The anti-angiogenic effect of miR-26a was mainly mediated by PI3K/Akt/HIF/VEGFA pathway in HCCLM3 cells. (C) After YC-1 or LY294002 was used to treat HCCLM3 cells with or without transfection of anti-miR-26a inhibitor, the expression of VEGFA was assessed by Western blot and ELISA. (D, E) After YC-1 or LY294002 was used to treat HCCLM3 cells, CM was collected and the effects of CM on HUVEC tube formation and migration were assessed. The tube length and the number of migration cells were evaluated by counting 10 random fields at ×100 magnification. * compare to WT-HCCLM3, P < 0.05; # compare to Control-HCCLM3, P < 0.05; ^ compare to anti-miR-26a-HCCLM3, P < 0.05. (TIF) [file pone.0077957.s004.tif]
