# Supplementary material for: MicroRNA-26a Inhibits Angiogenesis by Down-Regulating VEGFA through the PIK3C2α/Akt/HIF-1α Pathway in Hepatocellular Carcinoma
Source: PLoS One. 2013 Oct 23;8(10):e77957. doi: 10.1371/journal.pone.0077957 (PMC3806796; doi:10.1371/journal.pone.0077957)
Supplement: Table S1 — A total of 102 patients who underwent curative liver resection for pathology-proven HCC in our institute were enrolled. None of them received any preoperative anticancer treatment. These patients were observed between 1999 and 2003, and the clinicopathological features of them were shown. (DOC) [file pone.0077957.s005.doc]

**Table S1：**

Table 1 Clinicopathologic Features of the Study Patients

| Feature | No. of patients | % |
| --- | --- | --- |
| Age, years |  |  |
| Median | 51.29 |  |
| Range | 29–77 |  |
| Sex |  |  |
| Male | 88 | 86.3 |
| Female | 14 | 13.7 |
| α-fetoprotein, µg/ml |  |  |
| Median | 799.61 |  |
| Range | 1–5755 |  |
| Hepatitis B history |  |  |
| Yes | 89 | 97.3 |
| No | 13 | 127 |
| Liver cirrhosis | Missing : 11 | 10.8 |
| Yes | 78 | 76.5 |
| No | 13 | 12.7 |
| Hepatitis B e antigen |  |  |
| Positive | 22 | 21.6 |
| Negative | 80 | 78.4 |
| Tumor size, cm† |  |  |
| Median | 4.654 |  |
| Range | 1–15 |  |
| Tumor encapsulation |  |  |
| Complete | 50 | 49.0 |
| None | 52 | 51.0 |
| Microvascular invasion |  |  |
| Yes | 20 | 19.6 |
| No | 82 | 80.4 |
| Tumor differentiation |  |  |
| I-II | 71 | 69.6 |
| III-IV | 31 | 30.4 |
| TNM stage |  |  |
| I | 12 | 11.8 |
| II | 69 | 67.6 |
| IIIA | 21 | 20.6 |
| OS (months) |  |  |
| Median | 60.813 |  |
| Range | 2.0–144 |  |
| DFS (months) |  |  |
| Median | 40.098 |  |
| Range | 1–144 |  |
| miR-26a |  |  |
| Median | −3.1236 |  |
| Range | −6.16 to −0.63 |  |

OS, overall survival; DFS, disease-free survival.
